# Second solid cancers after radiotherapy for breast cancer in SEER cancer registries

**DOI:** 10.1038/sj.bjc.6605435

**Published:** 2009-11-24

**Authors:** A Berrington de Gonzalez, R E Curtis, E Gilbert, C D Berg, S A Smith, M Stovall, E Ron

**Affiliations:** 1 Radiation Epidemiology Branch, Division of Cancer Epidemiology and Genetics, National Cancer Institute, Bethesda, MD 20814, USA; 2 Early Detection Research Group, Division of Cancer Prevention, National Cancer Institute, Bethesda, MD 20814, USA; 3 Department of Radiation Physics, University of Texas M.D. Anderson Cancer Center, Houston, TX 70330, USA

**Keywords:** radiotherapy, second primary neoplasms, breast cancer, risk, radiation

## Abstract

**Background:**

Radiotherapy for breast cancer reduces disease recurrence and breast cancer mortality. However, it has also been associated with increased second cancer risks in exposed sites.

**Methods:**

We evaluated long-term second cancer risks among 182 057 5-year survivors of locoregional invasive breast cancer diagnosed between 1973 and 2000 and reported to US NCI-SEER Program cancer registries. Multivariate Poisson regression was used to estimate the relative risk (RR) and excess cases of second cancer in women who had surgery and radiotherapy, compared with those who had surgery alone. Second cancer sites were grouped according to doses received from typical tangential breast fields.

**Results:**

By the end of 2005 (median follow-up=13.0 years), 15 498 second solid cancers had occurred, including 6491 contralateral breast cancers. The RRs for radiotherapy were 1.45 (95% confidence interval (CI)=1.33–1.58) for high-dose second cancer sites (1+ Gy: lung, oesophagus, pleura, bone and soft tissue) and 1.09 (1.04–1.15) for contralateral breast cancer (≈1 Gy). These risks decreased with increasing age and year of treatment. There was no evidence of elevated risks for sites receiving medium (0.5–0.99 Gy, RR=0.89 (0.74–1.06)) or low doses (<0.5 Gy, RR=1.01 (0.95–1.07)). The estimated excess cases of cancer in women treated with radiotherapy were as follows: 176 (95% CI=69–284) contralateral breast cancers or 5% (2–8%) of the total in all 1+year survivors, and 292 (222–362) other solid cancers or 6% (4–7%) of the total.

**Conclusions:**

Most second solid cancers in breast cancer survivors are not related to radiotherapy.

Substantial improvements in breast cancer treatment and screening signify that nearly 90% of women diagnosed with breast cancer survive for >5 years (Berry *et al*, 2005; Ries *et al*, 2008). Currently, an estimated 2.5 million breast cancer survivors live in the United States (Ries *et al*, 2008), and the long-term health of these women is an important public health issue. A recent descriptive analysis of Surveillance Epidemiology and End Results Program (SEER) cancer registries found that breast cancer survivors have an 18% higher risk of developing a subsequent cancer compared with the general population (Curtis *et al*, 2006). Shared environmental and genetic factors are likely to be involved, but some of the increased risk is probably a late adverse effect from the cancer treatments such as radiotherapy (Clarke *et al*, 2005). Randomised trials and cancer registry-based studies have shown that radiotherapy significantly reduces the risk of recurrence and breast cancer mortality, and also increases the risk of second cancers of the lung, oesophagus, soft tissue, contralateral breast and leukaemia (Huang and Mackillop, 2001; Gao *et al*, 2003; Zablotska and Neugut, 2003; Clarke *et al*, 2005; Zablotska *et al*, 2005).

Surveillance Epidemiology and End Results Program registries cover the largest population of any national cancer registries and contain information on radiotherapy treatment, tumour characteristics and have long-term follow-up. In this study, we performed the first systematic evaluation of all second solid cancer risks after radiotherapy treatment for invasive breast cancer in the United States using the SEER databases. The large sample size and long-term follow-up enabled evaluation of patterns of effect modification, estimation of the number of excess cases and the proportion of second solid cancers occurring among breast cancer survivors that might be attributable to radiotherapy.

## Materials and methods

### Population and follow-up

The cohort was composed of women who were diagnosed with a first primary invasive locoregional breast cancer reported to one of the nine SEER registries between 1 January 1973 and 31 December 2000 (*n*=328 691). As there is at least a 5-year lag period between radiation exposure and solid cancer induction (BEIR VII, 2005), we excluded 103 235 women who did not survive for 5 years. The follow-up time for second cancers began 5 years after the date of initial cancer diagnosis and ended at the date of diagnosis of second primary cancer, at last known vital status, death or at the end of the study (31 December 2005), whichever occurred first. As underreporting of second cancers has been noted to occur among elderly patients who frequently have other comorbid conditions (Fraumeni *et al*, 2006), we excluded women whose first breast cancer was diagnosed after the age of 75 years (*n*=33 214) and restricted follow-up to attained ages <80 years. We also excluded women if it was unknown whether they had received radiotherapy (*n*=352), chemotherapy (*n*=1273) or hormonal therapy (*n*=1304); women who did not have breast cancer surgery or if surgery was unknown (*n*=4848) and women who had distant metastases at diagnosis or unknown disease stage (*n*=7853). As a number of women were missing more than one piece of information, the final cohort included 182 057 women.

### Treatment information

Women were classified according to whether they had received radiotherapy as part of their initial breast cancer treatment. Surgery was classified as mastectomy (total, modified radical, extended radical mastectomy) or breast-conserving surgery (partial mastectomy, lumpectomy, wedge resection, quadrantectomy, segmental mastectomy, tylectomy, subcutaneous mastectomy). Before 1983, the type of surgery performed was not available in SEER, but as breast-conserving surgery was rare, surgery before this date was assumed to have been mastectomy. The effects of chemotherapy and hormonal therapy were not assessed directly because of concerns regarding underascertainment in SEER (Brown *et al*, 2000), but the relative risks (RRs) for radiotherapy were adjusted for chemotherapy and hormonal therapy treatment.

We grouped second cancer sites *a priori* into three dose groups according to the estimated mean organ dose from the basic breast cancer radiotherapy treatment involving tangential fields: high (1+ Gy), medium (0.5–0.99 Gy) and low (<0.5 Gy) dose. This dosimetry was conducted using thermoluminscent dosimeters in tissue equivalent phantoms assuming a standard treatment protocol with 50 Gy tumour dose and beam energy of 6 MV photons (Appendix A) (Stovall *et al*, 2006; Stovall *et al*, 2008). Ipsilateral second breast cancers were excluded because of the difficulty in distinguishing between recurrence and subsequent primary cancer.

### Statistical analysis

The observed (O) number of second cancers for each site was compared with the expected (E) number in women in the general population. The expected number of second cancers was estimated by multiplying age-, sex-, race- and calendar-period-specific cancer incidence rates in the nine SEER registries by stratum-specific person-years at risk and then summing across strata (Seer^*^stat, version 6.4.4). Standardised incidence ratios (SIRs) were calculated by dividing the observed number of cancers (O) by the expected number (E). Tests of statistical significance were two sided and were based on Poisson exact methods (Liddell, 1984).

Multivariate Poisson regression analysis (using Epicure, 2007) was used to estimate the RR (and 95% confidence intervals (CI)) for the groups of second solid cancers (defined above according to dose) after radiotherapy and surgery compared with surgery alone, with the expected number of second cancers used as an offset. If *O*_*r*_ and *E*_*r*_ indicate the number of observed and expected cancers, respectively, in the surgery+radiotherapy (*r*=1) and surgery alone (*r*=0) groups, then the statistical expectation of *O*_*r*_ is given by 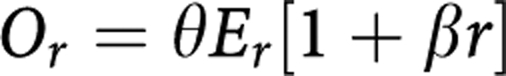 where *θ* and *β* are parameters to be estimated and 1+*β* is the RR associated with radiotherapy. The use of the expected number of cancers as an offset indirectly adjusts for potential confounding by attained age and attained calendar period (Yasui *et al*, 2003). Analyses were additionally adjusted for breast cancer stage, age at and year of breast cancer diagnosis, chemotherapy and hormonal therapy treatment (yes/no) through stratification. Trend tests were conducted using ordinal categories as continuous variables.

We investigated potential effect modifiers by stratification, including age at, year of and time since first cancer diagnosis, as well as stage and type of surgery (breast-conserving *vs* mastectomy). As the majority of women who receive breast-conserving surgery are recommended to receive radiotherapy, the proportion of women who had this type of surgery without radiotherapy in SEER was relatively small (17%), and this subgroup is unlikely to be a representative subset of breast cancer patients. Therefore, to obtain stable risk estimates in subgroup analyses according to surgery type, we used a combined surgery-only comparison group that included both breast-conserving surgery and mastectomy patients. Tests for heterogeneity between surgery types took account of the lack of independence of the comparison group (Berrington and Cox, 2003).

The number of excess cancers related to radiotherapy was estimated as the number of cases in those treated with radiotherapy minus the estimated number of cases that would have occurred in these women if they had not received radiotherapy. The proportion of second cancers attributable to radiotherapy was estimated by dividing this excess by the total number of second cancers in both 1+ and 5+ year survivors. Although it is unlikely that there are any radiation-related cancers before 5 years, the attributable risk among all survivors (defined here as 1+ years) is the most relevant summary statistic for all breast cancer survivors. The excess absolute risk (per 10 000 person-years) was estimated by dividing the excess by the associated number of person-years.

## Results

Overall, 38% of women with breast cancer had surgery+radiotherapy and 62% were treated with surgery alone (Table 1). Radiotherapy was slightly less common in those who were older at diagnosis, was increasingly common over time and was more frequent in those who received breast-conserving surgery. Within categories of surgery type, stage was related to treatment with radiotherapy, particularly after mastectomy, in which radiotherapy was more common after regional than after localised disease (27 *vs* 8%).

The women were followed up for an average of 13 years. During the follow-up period (1978–2005), a total of 15 498 women developed a second primary solid cancer, including 6491 contralateral breast cancers (Table 2). Women treated with surgery+radiotherapy had a higher risk of several second solid cancers compared with the general female population, with more than two-fold SIRs observed for oesophageal, pleural, bone, soft tissue and contralateral breast cancer. These sites typically receive doses from radiotherapy of 1 Gy or more (Appendix A). The adjusted RRs for surgery+radiotherapy, compared with surgery alone, were 1.09 (1.04–1.15) for contralateral breast cancer, 1.45 (95% CI=1.33–1.58) for high-dose, 0.89 (0.74–1.06) for medium-dose and 1.01 (0.95–1.07) for low-dose sites.

For sites in the high-dose group, the RR for surgery+radiotherapy, compared with surgery alone, decreased with increasing age at treatment (*P*-trend<0.001) and with increasing year of treatment (*P*-trend=0.01), but increased with increasing time since diagnosis (*P*-trend<0.001) (Table 3). For patients diagnosed with breast cancer in 1983 or later, the risks were higher after mastectomy (RR=1.50, 95% CI=1.22–1.82) than after breast-conserving surgery (RR=1.28, 95% CI=1.14–1.43), but the difference was not statistically significant (*P*>0.5).

We further examined the risks for specific sites within the high-dose group that had the largest number of cases: lung, oesophagus and soft tissue. The RR of lung cancer was significantly increased (RR=1.38 (95% CI=1.26–1.51)) and was higher for ipsilateral than for contralateral lung cancer (1.54 (1.36–1.75) *vs* 1.18 (1.02–1.35)). For patients diagnosed with breast cancer in 1983 or later, lung cancer risk was higher after mastectomy (RR=1.43 (95% CI=1.15–1.77)) than after breast-conserving surgery, but was still significantly elevated (RR=1.20 (1.07–1.36)). For oesophageal cancers, the RR was 1.99 (1.37–2.88), and there was no difference according to surgery type. Overall, the risk for soft tissue cancers was 2.52 (1.67–3.81), but it was especially highly elevated for angiosarcomas (RR=13.7 (4.0–95.6)). The majority of angiosarcomas diagnosed in women who had surgery+radiotherapy were located in the thorax (*n*=12 out of 16).

The RR for contralateral breast cancer in women who had surgery+radiotherapy, compared with those who had surgery alone, decreased with increasing age at first breast cancer treatment (*P*-trend=0.03, Table 4). The RR was lower for cancers treated after 1993 (*P*-trend=0.02), but was not clearly associated with time since treatment, stage or type of surgery.

In total, there were an estimated 176 (95% CI=69–284) excess cases of contralateral breast cancer in women who had surgery+radiotherapy, or 5% (95%CI=2–7%) of the contralateral breast cancers diagnosed in 1 year survivors (Table 5). The excess absolute risk of contralateral breast cancer associated with radiotherapy was 2 (1–4) cases per 10 000 person-years. There were an estimated 292 (95% CI=222–362) excess cases of solid cancers (other than contralateral breast cancer) in women who had radiotherapy, or 6% (3–8%) of the total in 1 year survivors (Table 5). The excess was largely composed of cancers of the lung (∼80%). The excess absolute risk for these cancers associated with radiotherapy was 4 (3–5) cases per 10 000 person-years. When considering only 5+year survivors, the attributable risk estimates were slightly higher: 8% (3–14%) for contralateral breast cancer and 10% (5–14%) for other solid cancers.

## Discussion

In this large long-term study of breast cancer survivors, significantly increased RRs of second solid cancers after radiotherapy were observed for the group of sites that typically receive the highest radiation exposure (⩾1 Gy). There was no overall excess risk for the group of sites that typically receive lower radiation exposures (<1 Gy). In total, it was estimated that about 5% (95% CI=2–7%) of contralateral breast cancers and 6% (3–8%) of other solid cancers occurring among 1+year survivors could be related to radiotherapy. Risks were generally lower for more recent treatment periods and higher for younger ages at treatment.

To our knowledge, this is the first study to assess and quantify the long-term risk of all solid cancers after breast cancer radiotherapy in the United States. Our site-specific results were broadly similar to those from a pooled analysis of 63 randomised clinical trials of breast cancer radiotherapy (Clarke *et al*, 2005) and several observational studies. In these studies, there was evidence of increased risks of lung (Zablotska and Neugut, 2003; Roychoudhuri *et al*, 2004; Clarke *et al*, 2005; Darby *et al*, 2005; Andersson *et al*, 2008; Schaapveld *et al*, 2008), oesophageal (Zablotska and Neugut, 2003; Roychoudhuri *et al*, 2004; Clarke *et al*, 2005) and contralateral breast cancer (Gao *et al*, 2003; Roychoudhuri *et al*, 2004; Clarke *et al*, 2005), as well as soft tissue sarcomas (Huang and Mackillop, 2001; Clarke *et al*, 2005; Andersson *et al*, 2008), in women treated with radiotherapy compared with unirradiated women. In the pooled analysis of clinical trials (Clarke *et al*, 2005), the authors did not estimate attributable risks, but it was possible to estimate them on the basis of the data presented. Overall, about 8% of total cancers were attributable to radiotherapy, slightly higher than the estimate from this study.

Our results suggested that, in general, radiation-related risks were lower among women treated in more recent years (1993+). This is likely the result of several factors, including reduced volume of irradiated tissues such as the lung, since the introduction of computed tomography planning (Muren *et al*, 2002; Wang and Harris, 2004; Van der Laan *et al*, 2008), and also because of shorter available follow-up time. Another factor that probably contributed to this trend was the increasing use of breast-conserving surgery. Although detailed treatment information was not available in SEER, women who received mastectomy probably received supraclavicular, and possibly also internal mammary irradiation, more often than women who had breast-conserving surgery, which would increase doses (Appendix A). In an earlier analysis of lung cancer risk after breast cancer radiotherapy in the SEER registries, it was suggested that there was no increased risk of lung cancer in women who had breast-conserving surgery (Zablotska *et al*, 2005). We used a combined comparison group of women who had surgery only (mastectomy or breast-conserving surgery) in the current analysis to obtain a broader, more representative referent group of women not treated with radiotherapy. Using this approach, there was evidence of a small, but significantly increased, risk of lung cancer and other cancers in high-dose regions after breast-conserving surgery. In terms of absolute numbers and fatality, lung cancer is by far the most important second cancer after breast cancer, hence it remains important to continue to monitor lung cancer risks after current radiation treatment protocols, taking into account smoking history (Prochazka *et al*, 2005; Kaufman *et al*, 2008).

Three earlier studies with individual dosimetry have reported an increased risk of contralateral breast cancer after radiotherapy in the youngest patients (<age 45) (Boice *et al*, 1992; Hooning *et al*, 2008; Stovall *et al*, 2008). This is consistent with the well-established modifying effects of age at radiation exposure; risk decreases with increasing age at exposure and is particularly low in post-menopausal women (Preston *et al*, 2002). This study and two earlier investigations (including the pooled analysis of clinical trials; Gao *et al*, 2003; Clarke *et al*, 2005) found an elevated risk of contralateral breast cancer after radiotherapy following young age at exposure, and also among post-menopausal women. Given the large number of post-menopausal breast cancer patients treated with radiotherapy, this is another question that warrants further study. Although we found no difference in contralateral breast cancer risk in women who had radiotherapy after mastectomy compared with those who underwent breast-conserving surgery, a recent Dutch study reported lower risks after mastectomy (Hooning *et al*, 2008). However, the majority of women in the Dutch study who had mastectomy were treated with electrons rather than with photons, which should result in a lower radiation dose to the contralateral breast (Stovall *et al*, 2008) and is the most likely explanation for the difference in findings.

There are several limitations in the use of SEER cancer registries to evaluate treatment-related second cancer risks. One limitation is the potential for loss to follow-up if a patient moves from the registry area, which would result in an underestimation of the absolute number of second cancers and hence the excess absolute risk estimates. However, mortality data are complete and comparisons of our results for lung cancer incidence with an earlier study of these women evaluating lung cancer mortality produced similar results, suggesting that this bias is likely to be small (Darby *et al*, 2005). Furthermore, the estimated attributable fraction should be valid, unless loss to follow-up is greater in one treatment group than in the other. Radiotherapy may be underreported in SEER because only information pertaining to the initial treatment course is collected (Malin *et al*, 2002). Since type of surgery was only recorded in SEER after 1983, we assumed that all breast cancer surgery before that time was mastectomy. This assumption undoubtedly resulted in misclassification, which most likely reduced the RR in the mastectomy group. Lack of individual data on actual treatment fields used also meant that we had to use general dose groupings based on typical organ doses from basic treatment fields. Even though individual organ doses would vary if additional fields were used, we think that it is unlikely that the rank order of organs, and hence our dose groupings, would change significantly. This can be observed in Appendix A for the use of tangential fields+supraclavicular fields.

As radiation treatment was not randomised, selection bias could result in differences between treatment groups with regard to smoking status and other variables that affect second cancer risk. Information on other potential confounding factors such as smoking is usually not available in cancer registry studies. The patterns of risk we observed were consistent with the general literature on radiation carcinogenesis, in that risks were higher for sites that should have received higher doses and also higher for younger ages at exposure (Preston *et al*, 2007). Furthermore, our findings were similar to the results from randomised clinical trials (Deutsch *et al*, 2003; Clarke *et al*, 2005), suggesting that any residual confounding is unlikely to have been substantial. If anything, residual confounding by smoking may have resulted in an underestimation of risks if smokers were less likely to receive radiotherapy than non-smokers.

The strengths of this study include the large size of the population-based cohort and long-term follow-up, which enabled the evaluation of relatively small RRs. We also conducted multivariate analyses to ensure that we controlled for the available confounding factors, including other breast cancer treatments and stage. This, plus the assessment of all second solid cancer sites in a single study, enabled the examination of effect modification by age and year of diagnosis, as well as estimation of the excess number and attributable proportion of second cancers that could be related to radiotherapy.

Most of the women in this study were treated in the past (<1990), and breast cancer treatment and radiotherapy techniques have changed considerably over the past three decades (Shank *et al*, 2000; Taylor *et al*, 2007). Indeed, our estimates of RRs were lower in those treated in more recent calendar years, and thus the combined results may overestimate risks for current treatment.

Overall, 5–6% of second solid cancers in irradiated women were estimated to be attributable to radiotherapy exposure. Among all breast cancer survivors, this figure was 3%. Our findings suggest that most second solid cancers after treatment for breast cancer are related to other risk factors such as lifestyle or genetic factors. When women and their physicians make treatment decisions, the risk of radiotherapy-related cancer needs to be placed in perspective and balanced with the known tumour control and mortality benefits achieved from treatment.

## Figures and Tables

**Table 1:** Descriptive statistics of the women who were diagnosed with a primary invasive locoregional breast cancer diagnosed before age 75 who survived 5 years (SEER 9 registries: 1973–2005)

	**Surgery+radiotherapy^a^**		**Surgery only^a^**		**Total**	
**Characteristic**	***n***	**%**	***n***	**%**	***n***	**%**
All women	69 296	38	112 761	62	182 057	100
						
*Age at treatment (diagnosis)*						
<40	6104	39	9511	61	15 615	100
40–49	17 075	41	24 711	59	41 786	100
50–59	20 056	40	30 047	60	50 103	100
60–69	18 534	36	33 460	64	51 994	100
70–74	7527	33	15 032	67	22 559	100
						
*Year of treatment (diagnosis)*						
1973–1982	8958	21	34 681	79	43 639	100
1983–1992	21 366	32	46 306	68	67 672	100
1993	38 972	55	31 774	45	70 746	100
						
*Stage (breast conserving surgery)*						
Localised	39 822	84	7503	16	47 325	100
Regional	10 456	81	2504	19	12 960	100
						
*Stage (mastectomy)*						
Localised	6216	8	67 834	92	74 050	100
Regional	12 802	27	34 920	73	47 722	100

a On the basis of initial treatment, chemotherapy and hormonal therapy were not considered in this classification.

Associations with radiotherapy *P*<0.001 for all variables listed above (calculated using multivariable logistic regression).

**Table 2:** Risk of second solid primary cancer after invasive locoregional breast cancer in 5-year survivors (SEER 9 registries: 1973–2005)

		**Surgery+radiotherapy**			**Surgery only**				
		**Observed**	**Expected**		**Observed**	**Expected**			
**Dose grouping^a^**	**Cancer site**	**cases**	**cases**	**SIR**	**cases**	**cases**	**SIR**	**RR^b^**	**(95% CI)**
High (1+ Gy)	Oesophagus	56	24.98	2.24^*^	68	61.58	1.10		
	Pleura	2	0.22	9.14^*^	0	0.50	0		
	Lung	814	673.16	1.21^*^	1,387	1582.33	0.88^*^		
	Bone	13	4.14	3.14^*^	17	9.33	1.82^*^		
	Soft tissue^c^	56	18.95	2.96^*^	48	42.50	1.13		
	*Sub-total*	941	721.50	1.30^*^	1520	1697.63	0.90^*^	1.45	(1.33–1.58)
									
Medium (0.5–0.99 Gy)	Stomach	56	54.18	1.02	158	138.36	1.14		
	Liver/gall bladder	35	61.90	0.57^*^	110	147.33	0.75^*^		
	Larynx	10	19.35	0.52^*^	35	47.27	0.74		
	Thyroid	72	62.78	1.15	129	122.43	1.05		
	CNS	4	2.76	1.45	8	6.13	1.31		
	*Sub-total*	177	200.98	0.88	440	461.75	0.95	0.89	(0.74–1.06)
									
Low (<0.5 Gy)	Oral cavity	61	64.74	0.94	147	158.72	0.93		
	Salivary gland	16	8.85	1.81^*^	24	20.26	1.18		
	Colon	364	387.89	0.94	921	975.15	0.94		
	Rectum	118	128.31	0.92	285	320.40	0.89		
	Pancreas	103	115.47	0.89	268	281.74	0.95		
	Melanoma of the skin	125	118.12	1.06	249	248.37	1.00		
	Cervix uteri	30	52.46	0.57^*^	75	124.08	0.60^*^		
	Ovary	219	152.42	1.43^*^	462	362.68	1.27^*^		
	Endometrial	421	301.52	1.40^*^	878	705.96	1.24^*^		
	Other female genital	33	37.45	0.88	80	88.47	0.90		
	Bladder	125	113.19	1.10	287	273.89	1.05		
	Kidney	71	85.30	0.83	170	191.37	0.89		
	Renal/other urinary tract	9	14.51	0.62	33	36.66	0.90		
	Brain	45	44.92	1.00	78	107.10	0.73^*^		
	Other sites	71	74.34	0.96	161	168.79	0.95		
	*Sub-total*	1811	1699.50	1.07^*^	4118	4063.64	1.01	1.01	(0.95–1.07)
									
All solid cancers (excluding contralateral breast)		2929	2621.98	1.12^*^	6078	6223.02	0.98	1.11	(1.06–1.16)
Contralateral breast		2076	688.07	3.02^*^	4415	1571.94	2.81^*^	1.09	(1.04–1.15)

Abbreviations: CI=confidence interval; CNS=central nervous system; SIR=standardised incidence ratio=ratio of observed to expected cases.

a Mean doses on the basis of tangential fields breast radiotherapy, see Table 1. ^*^P<0.05.

b RR=relative risk calculated using Poisson regression stratified by stage, age at treatment, year of treatment, chemotherapy and hormonal therapy.

c Soft tissue histology: surgery+radiotherapy includes 16 angiosarcomas, 22 fibrosarcomas, 18 others and surgery only includes 2 angiosarcomas, 18 fibrosarcomas and 28 others.

**Table 3:** Risk of subsequent primary solid cancer at highly exposed sites (>1 Gy: oesophagus, pleura, lung, bone, connective tissue) after invasive locoregional breast cancer in 5-year survivors (SEER 9 registries: 1973–2005)

	**Surgery+radiotherapy**			**Surgery only**					
**Characteristic**	**Observed**	**Expected**	**SIR**	**Observed**	**Expected**	**SIR**	**RR^a^**	**(95% CI)**	**P-trend/homogeneity**
*Age at diagnosis*									
<40	45	17.33	2.60	50	47.07	1.06	2.67	(1.75–4.07)	
40–49	195	119.86	1.63	310	318.74	0.97	1.67	(1.38–2.02)	
50–59	310	251.87	1.23	542	625.41	0.87	1.40	(1.21–1.62)	
60+	391	332.45	1.18	618	706.41	0.87	1.31	(1.15–1.50)	<0.001
									
*Year of diagnosis*									
1973–1982	268	168.17	1.59	646	735.03	0.88	1.77	(1.52–2.05)	
1983–1992	415	336.02	1.24	672	763.30	0.88	1.40	(1.24–1.59)	
1993+	258	217.30	1.19	202	199.30	1.01	1.15	(0.95–1.38)	0.01
									
*Latency*									
5–9 years	488	406.05	1.20	685	750.51	0.91	1.30	(1.15–1.47)	
10–14 years	268	190.88	1.40	455	485.07	0.94	1.51	(1.30–1.77)	
15+ years	185	124.57	1.49	380	462.05	0.82	1.80	(1.50–2.16)	<0.001
									
*Disease stage*									
Localised	594	484.66	1.23	1051	1187.53	0.89	1.40	(1.25–1.55)	
Regional	347	236.85	1.47	469	510.01	0.92	1.55	(1.35–1.80)	0.24
									
*Surgery (1980+)* b									
Breast conserving	550	467.39	1.18	874	962.6	0.91	1.28	(1.14–1.43)	
Mastectomy	123	85.94	1.43				1.50	(1.22–1.82)	>0.5^c^

Abbreviations: CI=confidence interval; SIR=standardised incidence ratio=ratio of observed to expected cancers.

a RR=relative risk calculated using Poisson regression with stratification by stage, age at treatment, year of treatment, chemotherapy and hormonal therapy.

b Comparison group of surgery only was on the basis of breast conserving surgery and mastectomy combined.

c Estimated using methods that account for shared comparison group (Berrington and Cox, 2003).

**Table 4:** Risk of contralateral breast cancer after invasive locoregional breast cancer in 5-year survivors (SEER 9 1973–2005)

	**Surgery+radiotherapy**			**Surgery only**					
**Characteristic**	**Observed**	**Expected**	**SIR**	**Observed**	**Expected**	**SIR**	**RR^a^**	**(95% CI)**	**P-trend/homogeneity**
*Age at diagnosis*									
<40	277	41.05	6.75	490	97.15	5.04	1.30	(1.11–1.50)	
40–49	517	166.07	3.11	1089	377.01	2.89	1.08	(0.97–1.20)	
50–59	598	233.21	2.56	1437	542.25	2.65	0.98	(0.89–1.08)	
60+	684	247.74	2.76	1399	555.53	2.52	1.14	(1.04–1.26)	0.03
									
*Year of diagnosis*									
1975–1982	557	160.34	3.47	2083	685.68	3.04	1.12	(1.02–1.23)	
1983–1992	964	318.28	3.03	1849	699.15	2.64	1.14	(1.05–1.23)	
1993+	555	209.46	2.65	483	187.11	2.58	1.04	(0.92–1.18)	0.02
									
*Latency*									
5–9 years	1233	401.61	3.07	2194	736.14	2.98	1.06	(0.99–1.14)	
10–14 years	554	179.78	3.08	1236	450.07	2.75	1.12	(1.01–1.24)	
15+ years	289	106.68	2.71	985	385.73	2.55	1.04	(0.91–1.19)	0.1
									
*Disease stage*									
Localised	1337	454.98	2.94	2988	1088.40	2.75	1.10	(1.03–1.18)	
Regional	739	233.09	3.17	1427	483.54	2.95	1.08	(0.98–1.18)	>0.5
									
*Surgery type (1980+)* b									
Breast conserving	1256	442.25	2.84	2332	886.26	2.63	1.10	(1.03–1.18)	
Mastectomy	263	85.49	3.08				1.11	(0.97–1.26)	>0.5^c^

Abbreviations: CI=confidence interval; SIR=standardised incidence ratio=ratio of observed to expected cancers.

a RR=relative risk calculated for treatment with surgery+radiotherapy compared with surgery alone using Poisson regression with stratification by stage, age at treatment, year of treatment, chemotherapy and hormonal therapy. Women with bilateral breast cancer at diagnosis or unknown laterality were excluded.

b Comparison group of surgery only was on the basis of breast conserving surgery and mastectomy combined.

c Calculated using methods to account for the shared comparison group (Berrington and Cox, 2003).

**Table 5:** Estimated number of excess solid cancers, attributable risk and excess absolute risk (EAR) per 10 000 person-years related to radiotherapy in those treated with surgery+radiotherapy for invasive locoregional breast cancer^a^ (SEER 9 registries: 1973–2005)

		**Excess cancers**		**Attributable risk**		**EAR/10 000 P-Y**	
	**Total second cancers**	***n***	**(95% CI)**	**%**	**(95% CI)**	***n***	**(95% CI)**
*Contralateral breast cancer*							
5+-year survivors (RT+surgery)	2076	176	(69–284)	8	(3–14%)	5	(2–7)
1+-year survivors (RT+surgery)	3775	176	(69–284)	5	(2–8%)	2	(1–4)
							
*All other solid cancers*							
5+-year survivors (RT+surgery)	2929	292	(222–362)	10	(5–14%)	8	(6–9)
1+-year survivors (RT+surgery)	5089	292	(222–362)	6	(4–7%)	4	(3–5)

Abbreviations: CI, confidence interval; P–Y=person-years; RT=radiotherapy.

a Analyses assume a 5+-year minimum latent period for radiation-related solid cancers and, therefore, no excess cancers related to radiation would occur in the 1–5-year interval. Therefore, only the denominator (total second cancers) changes in the two analyses.

**Table A1:** Estimated typical organ doses from breast radiotherapy assuming 50 Gy tumour dose and 6 MV photon beam energy^a^

		**Mean dose (range) (Gy)**	
**Dose grouping^b^**	**Cancer site**	**Tangential breast fields**	**Tangential breast fields+supraclavicular**
High	Oesophagus	1.1	5.6 (1.2–19.0)
	Pleura	n.a.	n.a.
	Lung (ipsilateral/contralateral)	5.7 (0.8–42)/0.9	10.0 (1.4–42)/1.1
	Bone	(0.03–50.0)	(0.5–50.0)
	Connective tissue (heart)	2.2 (0.8–5.5)	2.5 (1.0–6.0)
			
Medium	Stomach	0.5	0.5
	Liver	0.9 (0.3–3.0)	0.9 (0.3–3.0)
	Gall bladder	0.8	0.9
	Larynx	0.6	3.2 (2.0–5.0)
	Thyroid	0.9	10.0 (6.0–25.0)
	CNS	(0.2–1.0)	(0.3–19.0)
			
Low	Oral cavity	(0.2–0.4)	(0.5–1.3)
	Salivary gland	0.3	0.8
	Colon	0.2	0.2
	Rectum	0.06	0.07
	Pancreas	0.48	0.5
	Melanoma of the skin	n.a.	n.a.
	Cervix uteri	0.06	0.1
	Ovary	0.07	0.1
	Endometrial	0.06	0.1
	Other female genital	0.05	0.1
	Bladder	0.05	0.1
	Kidney	0.45	0.5
	Renal/other urinary tract	0.45	0.5
	Brain	0.1	0.2
	Other sites	<0.5	<0.5
			
Contralateral breast		1.0 (0.6–4.0)	1.2 (0.8–4.4)

Abbreviations: CNS=central nervous system; n.a.=not available.

Ipsilateral and contralateral lung, respectively.

a Addition of an electron beam boost field was also considered, but this did not change the dose category for the second cancer as the additional scatter doses were minimal.

b Dose grouping used in the paper: high (>1 Gy), medium (0.5–0.99 Gy) and low (<0.5 Gy) are on the basis of tangential breast fields.

## Appendix A

Table A1
